# 9,9-Dibutyl-9*H*-fluorene-2-carbonitrile

**DOI:** 10.1107/S1600536812022672

**Published:** 2012-05-26

**Authors:** Dong-dong Zhao, Peng Jiang, Hong-Jun Zhu

**Affiliations:** a Department of Chemical Engineering, College of Science, Nanjing University of Technology, Nanjing 210009, People’s Republic of China; b Department of Organic Chemistry, College of Science, Nanjing University of Technology, Nanjing 210009, People’s Republic of China; c Department of Applied Chemistry, College of Science, Nanjing University of Technology, Nanjing 210009, People’s Republic of China

## Abstract

The fluorene fragment of the title compound, C_22_H_25_N, is essentially planar, with an r.m.s deviation of the five-membered ring of 0.005 (2) Å. The dihedral angle between this ring and the outer benzene rings are 1.5 (2) and 0.7 (2)° while that between the benzene rings is 2.1 (2)°. The cyano group makes an angle of 0.3 (2)° with the attached benzene ring.

## Related literature

For applications of the title compound, including as a substrate in the synthesis of organic light-emitting materials, see: Jiang *et al.* (2012 ▶). For its synthesis, see: Omer *et al.* (2010 ▶). For bond-length data, see: Allen *et al.* (1987 ▶).
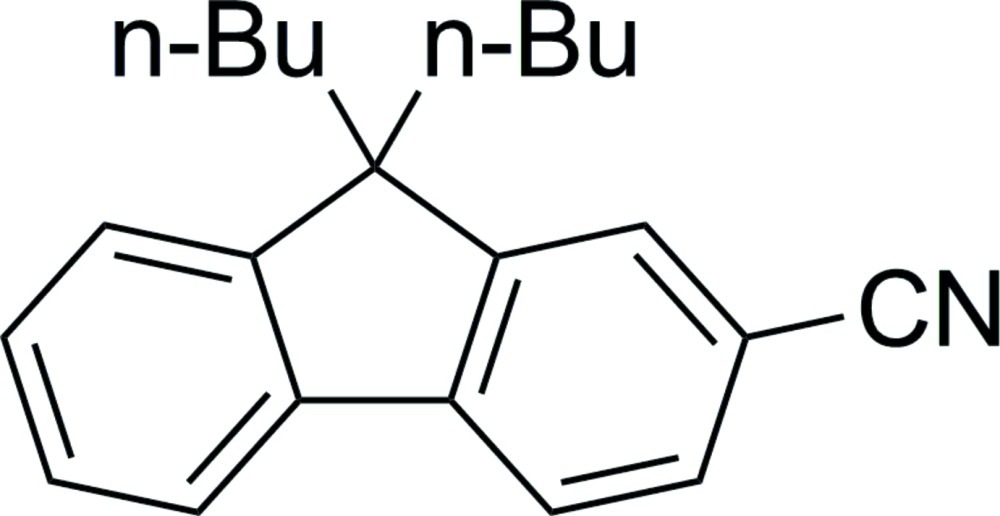


## Experimental

### 

#### Crystal data

C_22_H_25_N
*M* *_r_* = 303.43
Triclinic, 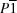

*a* = 9.2810 (19) Å
*b* = 9.994 (2) Å
*c* = 11.885 (2) Å
α = 100.35 (3)°
β = 96.73 (3)°
γ = 117.42 (3)°
*V* = 937.0 (3) Å^3^
*Z* = 2
Mo *K*α radiation
μ = 0.06 mm^−1^
*T* = 293 K
0.30 × 0.20 × 0.10 mm

#### Data collection

Enraf–Nonius CAD-4 diffractometer
Absorption correction: ψ scan (North *et al.*, 1968 ▶) *T*_min_ = 0.982, *T*_max_ = 0.994
3652 measured reflections
3420 independent reflections
1875 reflections with *I* > 2σ(*I*)
*R*_int_ = 0.027
3 standard reflections every 200 reflections intensity decay: 1%

#### Refinement

*R*[*F* ^2^ > 2σ(*F* ^2^)] = 0.068
*wR*(*F* ^2^) = 0.186
*S* = 1.00
3420 reflections
208 parameters
1 restraint
H-atom parameters constrained
Δρ_max_ = 0.45 e Å^−3^
Δρ_min_ = −0.13 e Å^−3^

### 

Data collection: *CAD-4 EXPRESS* (Enraf–Nonius, 1994 ▶); cell refinement: *SET4* in *CAD-4 EXPRESS*; data reduction: *XCAD4* (Harms & Wocadlo, 1995 ▶); program(s) used to solve structure: *SHELXS97* (Sheldrick, 2008 ▶); program(s) used to refine structure: *SHELXL97* (Sheldrick, 2008 ▶); molecular graphics: *SHELXTL* (Sheldrick, 2008 ▶); software used to prepare material for publication: *SHELXTL*.

## Supplementary Material

Crystal structure: contains datablock(s) I, global. DOI: 10.1107/S1600536812022672/im2373sup1.cif

Structure factors: contains datablock(s) I. DOI: 10.1107/S1600536812022672/im2373Isup2.hkl

Supplementary material file. DOI: 10.1107/S1600536812022672/im2373Isup3.cml

Additional supplementary materials:  crystallographic information; 3D view; checkCIF report

## Figures and Tables

**Fig. 1.**
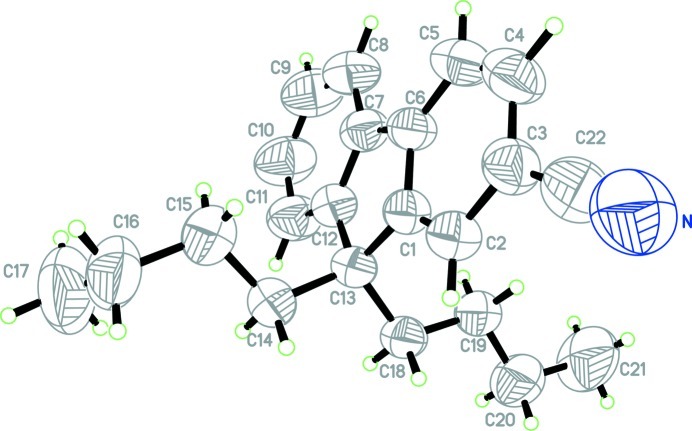
The molecular structure of (I) with displacement ellipsoids drawn at the 30% probability level.

## supplementary crystallographic information

### Comment

The title compound, 2-cyano-9,9-dibutylfluorene, is an important compound which can be used in many fields such as a substrate in the synthesis of organic light-emitting materials (Jiang *et al.*, 2012). We report here the crystal structure of the title compound, (I).

The molecular structure of (I) is shown in Fig. 1. Bond lengths are within normal ranges (Allen *et al.*, 1987).

In the molecule of the title compound, the fluorene fragment of the title compound, C_22_H_25_N, is essentially planar with a r.m.s deviation of ring A (C1/C6/C7/C12/C13) of 0.005 (2). The dihedral angle between ring A (C1/C6/C7/C12/C13) and the benzene rings B (C1—C6) and C (C7—C12) is 1.5 (2)° and 0.7 (2)°, respectively. The dihedral angle between the benzene rings B and C is 2.1 (2)°. The angle of cyano group with the benzene ring B is 0.3 (2)°.

### Experimental

The title compound,(I) was prepared according to the literature method (Omer *et al.*, 2010). Yellow block-shaped crystals were obtained by dissolving (I) (0.5 g, 1.04 mmol) in a mixed solution (10 ml petroleum ether and 1 ml EtOAc) and evaporating the solvent slowly at room temperature for about 5 d.

### Refinement

All hydrogen atoms were positioned geometrically, with C—H = 0.93 Å for aromatic H, and constrained to ride on their parent atoms, with *U*_iso_(H) = *xU*_eq_ (C), where *x* = 1.2 for aromatic H, and *x* = 1.5 for other H.

### Crystal data

**Table:**

C_22_H_25_N	*Z* = 2
*M**_r_* = 303.43	*F*(000) = 328
Triclinic, *P*1	*D*_x_ = 1.075 Mg m^−^^3^
Hall symbol: -P 1	Melting point: 374 K
*a* = 9.2810 (19) Å	Mo *K*α radiation, λ = 0.71073 Å
*b* = 9.994 (2) Å	Cell parameters from 25 reflections
*c* = 11.885 (2) Å	θ = 9–13°
α = 100.35 (3)°	µ = 0.06 mm^−^^1^
β = 96.73 (3)°	*T* = 293 K
γ = 117.42 (3)°	Block, yellow
*V* = 937.0 (3) Å^3^	0.30 × 0.20 × 0.10 mm

### Data collection

**Table:**

Enraf–Nonius CAD-4 diffractometer	1875 reflections with *I* > 2σ(*I*)
Radiation source: fine-focus sealed tube	*R*_int_ = 0.027
Graphite monochromator	θ_max_ = 25.4°, θ_min_ = 1.8°
ω/2θ scans	*h* = 0→11
Absorption correction: ψ scan (North *et al.*, 1968)	*k* = −12→10
*T*_min_ = 0.982, *T*_max_ = 0.994	*l* = −14→14
3652 measured reflections	3 standard reflections every 200 reflections
3420 independent reflections	intensity decay: 1%

### Refinement

**Table:**

Refinement on *F*^2^	Primary atom site location: structure-invariant direct methods
Least-squares matrix: full	Secondary atom site location: difference Fourier map
*R*[*F*^2^ > 2σ(*F*^2^)] = 0.068	Hydrogen site location: inferred from neighbouring sites
*wR*(*F*^2^) = 0.186	H-atom parameters constrained
*S* = 1.00	*w* = 1/[σ^2^(*F*_o_^2^) + (0.070*P*)^2^ + 0.3*P*] where *P* = (*F*_o_^2^ + 2*F*_c_^2^)/3
3420 reflections	(Δ/σ)_max_ < 0.001
208 parameters	Δρ_max_ = 0.45 e Å^−^^3^
1 restraint	Δρ_min_ = −0.13 e Å^−^^3^

### Special details

**Table:**

Geometry. All e.s.d.'s (except the e.s.d. in the dihedral angle between two l.s. planes) are estimated using the full covariance matrix. The cell e.s.d.'s are taken into account individually in the estimation of e.s.d.'s in distances, angles and torsion angles; correlations between e.s.d.'s in cell parameters are only used when they are defined by crystal symmetry. An approximate (isotropic) treatment of cell e.s.d.'s is used for estimating e.s.d.'s involving l.s. planes.
Refinement. Refinement of *F*^2^ against ALL reflections. The weighted *R*-factor *wR* and goodness of fit *S* are based on *F*^2^, conventional *R*-factors *R* are based on *F*, with *F* set to zero for negative *F*^2^. The threshold expression of *F*^2^ > σ(*F*^2^) is used only for calculating *R*-factors(gt) *etc*. and is not relevant to the choice of reflections for refinement. *R*-factors based on *F*^2^ are statistically about twice as large as those based on *F*, and *R*- factors based on ALL data will be even larger.

### Fractional atomic coordinates and isotropic or equivalent isotropic displacement parameters (Å^2^)

**Table:**

	*x*	*y*	*z*	*U*_iso_*/*U*_eq_	
N	−0.2377 (4)	−0.2430 (4)	0.3798 (3)	0.1162 (11)	
C1	0.2296 (3)	0.0884 (3)	0.2410 (2)	0.0587 (7)	
C2	0.1181 (3)	0.0220 (3)	0.3086 (2)	0.0643 (7)	
H2A	0.1403	0.0698	0.3881	0.077*	
C3	−0.0281 (3)	−0.1178 (3)	0.2548 (3)	0.0692 (7)	
C4	−0.0608 (4)	−0.1901 (3)	0.1362 (3)	0.0816 (9)	
H4A	−0.1594	−0.2834	0.1014	0.098*	
C5	0.0513 (4)	−0.1248 (3)	0.0704 (3)	0.0782 (9)	
H5A	0.0301	−0.1742	−0.0086	0.094*	
C6	0.1966 (3)	0.0153 (3)	0.1223 (2)	0.0624 (7)	
C7	0.3335 (3)	0.1124 (3)	0.0740 (2)	0.0658 (7)	
C8	0.3608 (4)	0.0907 (4)	−0.0388 (3)	0.0875 (9)	
H8A	0.2854	0.0015	−0.0980	0.105*	
C9	0.5023 (5)	0.2045 (5)	−0.0604 (3)	0.1045 (12)	
H9A	0.5215	0.1928	−0.1357	0.125*	
C10	0.6160 (5)	0.3356 (4)	0.0274 (3)	0.1079 (12)	
H10A	0.7116	0.4102	0.0110	0.130*	
C11	0.5895 (4)	0.3572 (4)	0.1397 (3)	0.0883 (10)	
H11A	0.6659	0.4461	0.1988	0.106*	
C12	0.4472 (3)	0.2443 (3)	0.1626 (2)	0.0652 (7)	
C13	0.3936 (3)	0.2420 (3)	0.2785 (2)	0.0587 (7)	
C14	0.5202 (3)	0.2379 (3)	0.3724 (2)	0.0684 (8)	
H14A	0.6249	0.3348	0.3892	0.082*	
H14B	0.4798	0.2341	0.4441	0.082*	
C15	0.5545 (4)	0.1028 (4)	0.3405 (3)	0.0891 (10)	
H15A	0.5793	0.0965	0.2633	0.107*	
H15B	0.4544	0.0062	0.3359	0.107*	
C16	0.6984 (5)	0.1167 (5)	0.4276 (3)	0.1128 (13)	
H16A	0.6845	0.1435	0.5066	0.135*	
H16B	0.6929	0.0156	0.4143	0.135*	
C17	0.8637 (6)	0.2332 (7)	0.4198 (5)	0.166 (2)	
H17A	0.9473	0.2382	0.4789	0.248*	
H17B	0.8703	0.3337	0.4319	0.248*	
H17C	0.8819	0.2042	0.3435	0.248*	
C18	0.3682 (3)	0.3808 (3)	0.3257 (2)	0.0625 (7)	
H18A	0.3309	0.3698	0.3981	0.075*	
H18B	0.4755	0.4756	0.3451	0.075*	
C19	0.2465 (4)	0.4010 (3)	0.2449 (2)	0.0739 (8)	
H19A	0.1378	0.3081	0.2269	0.089*	
H19B	0.2819	0.4109	0.1718	0.089*	
C20	0.2307 (5)	0.5418 (4)	0.2962 (3)	0.1006 (11)	
H20A	0.2037	0.5355	0.3722	0.121*	
H20B	0.3378	0.6351	0.3089	0.121*	
C21	0.1008 (6)	0.5581 (6)	0.2209 (4)	0.1452 (17)	
H21A	0.0981	0.6498	0.2590	0.218*	
H21B	−0.0064	0.4676	0.2095	0.218*	
H21C	0.1279	0.5672	0.1461	0.218*	
C22	−0.1465 (4)	−0.1884 (4)	0.3242 (3)	0.0849 (9)	

### Atomic displacement parameters (Å^2^)

**Table:**

	*U*^11^	*U*^22^	*U*^33^	*U*^12^	*U*^13^	*U*^23^
N	0.092 (2)	0.094 (2)	0.125 (3)	0.0158 (18)	0.039 (2)	0.0225 (19)
C1	0.0596 (16)	0.0563 (15)	0.0570 (15)	0.0306 (13)	0.0061 (13)	0.0067 (12)
C2	0.0649 (17)	0.0584 (16)	0.0611 (16)	0.0283 (15)	0.0085 (14)	0.0062 (13)
C3	0.0641 (17)	0.0559 (16)	0.079 (2)	0.0258 (14)	0.0094 (14)	0.0128 (14)
C4	0.074 (2)	0.0562 (17)	0.087 (2)	0.0209 (16)	−0.0050 (18)	0.0046 (16)
C5	0.087 (2)	0.0663 (18)	0.0619 (17)	0.0320 (17)	0.0012 (16)	0.0007 (14)
C6	0.0681 (17)	0.0559 (15)	0.0562 (16)	0.0317 (14)	0.0023 (13)	0.0031 (13)
C7	0.0797 (19)	0.0696 (18)	0.0539 (16)	0.0437 (16)	0.0139 (14)	0.0105 (14)
C8	0.107 (3)	0.083 (2)	0.0657 (19)	0.046 (2)	0.0185 (18)	0.0053 (16)
C9	0.134 (3)	0.107 (3)	0.076 (2)	0.058 (3)	0.047 (2)	0.022 (2)
C10	0.118 (3)	0.100 (3)	0.097 (3)	0.042 (2)	0.053 (2)	0.021 (2)
C11	0.084 (2)	0.084 (2)	0.080 (2)	0.0303 (19)	0.0287 (18)	0.0086 (17)
C12	0.0658 (17)	0.0667 (17)	0.0613 (16)	0.0340 (15)	0.0142 (14)	0.0091 (14)
C13	0.0553 (15)	0.0584 (15)	0.0524 (14)	0.0254 (13)	0.0067 (12)	0.0032 (12)
C14	0.0605 (16)	0.0752 (18)	0.0640 (17)	0.0334 (15)	0.0085 (13)	0.0092 (14)
C15	0.093 (2)	0.094 (2)	0.093 (2)	0.060 (2)	0.0151 (19)	0.0181 (18)
C16	0.121 (3)	0.146 (4)	0.112 (3)	0.097 (3)	0.022 (2)	0.039 (3)
C17	0.101 (3)	0.239 (6)	0.176 (5)	0.105 (4)	0.022 (3)	0.048 (4)
C18	0.0583 (16)	0.0606 (16)	0.0571 (15)	0.0246 (13)	0.0107 (13)	0.0043 (12)
C19	0.0756 (19)	0.0799 (19)	0.0663 (17)	0.0441 (17)	0.0084 (15)	0.0081 (15)
C20	0.116 (3)	0.099 (3)	0.104 (3)	0.073 (2)	0.017 (2)	0.014 (2)
C21	0.178 (4)	0.184 (5)	0.137 (4)	0.143 (4)	0.024 (3)	0.043 (3)
C22	0.074 (2)	0.064 (2)	0.095 (2)	0.0224 (17)	0.0118 (18)	0.0086 (17)

### Geometric parameters (Å, º)

**Table:**

N—C22	1.135 (4)	C13—C14	1.544 (3)
C1—C2	1.382 (3)	C14—C15	1.519 (4)
C1—C6	1.393 (3)	C14—H14A	0.9700
C1—C13	1.522 (3)	C14—H14B	0.9700
C2—C3	1.390 (4)	C15—C16	1.525 (4)
C2—H2A	0.9300	C15—H15A	0.9700
C3—C4	1.390 (4)	C15—H15B	0.9700
C3—C22	1.447 (4)	C16—C17	1.464 (5)
C4—C5	1.366 (4)	C16—H16A	0.9700
C4—H4A	0.9300	C16—H16B	0.9700
C5—C6	1.384 (4)	C17—H17A	0.9600
C5—H5A	0.9300	C17—H17B	0.9600
C6—C7	1.457 (4)	C17—H17C	0.9600
C7—C12	1.385 (4)	C18—C19	1.505 (4)
C7—C8	1.391 (4)	C18—H18A	0.9700
C8—C9	1.376 (4)	C18—H18B	0.9700
C8—H8A	0.9300	C19—C20	1.508 (4)
C9—C10	1.376 (5)	C19—H19A	0.9700
C9—H9A	0.9300	C19—H19B	0.9700
C10—C11	1.383 (4)	C20—C21	1.504 (5)
C10—H10A	0.9300	C20—H20A	0.9700
C11—C12	1.383 (4)	C20—H20B	0.9700
C11—H11A	0.9300	C21—H21A	0.9600
C12—C13	1.519 (3)	C21—H21B	0.9600
C13—C18	1.531 (3)	C21—H21C	0.9600
			
C2—C1—C6	120.5 (2)	C13—C14—H14B	108.3
C2—C1—C13	128.1 (2)	H14A—C14—H14B	107.4
C6—C1—C13	111.4 (2)	C14—C15—C16	113.8 (3)
C1—C2—C3	118.4 (2)	C14—C15—H15A	108.8
C1—C2—H2A	120.8	C16—C15—H15A	108.8
C3—C2—H2A	120.8	C14—C15—H15B	108.8
C2—C3—C4	120.9 (3)	C16—C15—H15B	108.8
C2—C3—C22	119.0 (3)	H15A—C15—H15B	107.7
C4—C3—C22	120.1 (3)	C17—C16—C15	114.2 (3)
C5—C4—C3	120.3 (3)	C17—C16—H16A	108.7
C5—C4—H4A	119.8	C15—C16—H16A	108.7
C3—C4—H4A	119.8	C17—C16—H16B	108.7
C4—C5—C6	119.5 (3)	C15—C16—H16B	108.7
C4—C5—H5A	120.3	H16A—C16—H16B	107.6
C6—C5—H5A	120.3	C16—C17—H17A	109.5
C5—C6—C1	120.3 (3)	C16—C17—H17B	109.5
C5—C6—C7	131.4 (3)	H17A—C17—H17B	109.5
C1—C6—C7	108.3 (2)	C16—C17—H17C	109.5
C12—C7—C8	120.8 (3)	H17A—C17—H17C	109.5
C12—C7—C6	108.5 (2)	H17B—C17—H17C	109.5
C8—C7—C6	130.7 (3)	C19—C18—C13	116.2 (2)
C9—C8—C7	118.3 (3)	C19—C18—H18A	108.2
C9—C8—H8A	120.9	C13—C18—H18A	108.2
C7—C8—H8A	120.9	C19—C18—H18B	108.2
C8—C9—C10	121.2 (3)	C13—C18—H18B	108.2
C8—C9—H9A	119.4	H18A—C18—H18B	107.4
C10—C9—H9A	119.4	C18—C19—C20	113.2 (2)
C9—C10—C11	120.6 (3)	C18—C19—H19A	108.9
C9—C10—H10A	119.7	C20—C19—H19A	108.9
C11—C10—H10A	119.7	C18—C19—H19B	108.9
C12—C11—C10	118.8 (3)	C20—C19—H19B	108.9
C12—C11—H11A	120.6	H19A—C19—H19B	107.7
C10—C11—H11A	120.6	C21—C20—C19	114.2 (3)
C11—C12—C7	120.3 (3)	C21—C20—H20A	108.7
C11—C12—C13	128.0 (2)	C19—C20—H20A	108.7
C7—C12—C13	111.7 (2)	C21—C20—H20B	108.7
C12—C13—C1	100.2 (2)	C19—C20—H20B	108.7
C12—C13—C18	112.8 (2)	H20A—C20—H20B	107.6
C1—C13—C18	111.7 (2)	C20—C21—H21A	109.5
C12—C13—C14	111.2 (2)	C20—C21—H21B	109.5
C1—C13—C14	111.3 (2)	H21A—C21—H21B	109.5
C18—C13—C14	109.4 (2)	C20—C21—H21C	109.5
C15—C14—C13	115.9 (2)	H21A—C21—H21C	109.5
C15—C14—H14A	108.3	H21B—C21—H21C	109.5
C13—C14—H14A	108.3	N—C22—C3	179.1 (4)
C15—C14—H14B	108.3		
			
C6—C1—C2—C3	−0.8 (4)	C8—C7—C12—C13	−179.0 (3)
C13—C1—C2—C3	177.7 (3)	C6—C7—C12—C13	1.0 (3)
C1—C2—C3—C4	0.6 (4)	C11—C12—C13—C1	179.9 (3)
C1—C2—C3—C22	−179.7 (3)	C7—C12—C13—C1	−0.8 (3)
C2—C3—C4—C5	0.3 (4)	C11—C12—C13—C18	61.0 (4)
C22—C3—C4—C5	−179.4 (3)	C7—C12—C13—C18	−119.7 (2)
C3—C4—C5—C6	−1.0 (4)	C11—C12—C13—C14	−62.3 (4)
C4—C5—C6—C1	0.8 (4)	C7—C12—C13—C14	117.0 (3)
C4—C5—C6—C7	−178.0 (3)	C2—C1—C13—C12	−178.5 (3)
C2—C1—C6—C5	0.1 (4)	C6—C1—C13—C12	0.2 (3)
C13—C1—C6—C5	−178.7 (2)	C2—C1—C13—C18	−58.7 (3)
C2—C1—C6—C7	179.2 (2)	C6—C1—C13—C18	119.9 (2)
C13—C1—C6—C7	0.4 (3)	C2—C1—C13—C14	63.9 (3)
C5—C6—C7—C12	178.1 (3)	C6—C1—C13—C14	−117.5 (2)
C1—C6—C7—C12	−0.9 (3)	C12—C13—C14—C15	−56.8 (3)
C5—C6—C7—C8	−1.9 (5)	C1—C13—C14—C15	54.0 (3)
C1—C6—C7—C8	179.1 (3)	C18—C13—C14—C15	177.9 (2)
C12—C7—C8—C9	−0.9 (5)	C13—C14—C15—C16	171.4 (3)
C6—C7—C8—C9	179.1 (3)	C14—C15—C16—C17	−74.2 (4)
C7—C8—C9—C10	1.2 (5)	C12—C13—C18—C19	56.3 (3)
C8—C9—C10—C11	−1.0 (6)	C1—C13—C18—C19	−55.7 (3)
C9—C10—C11—C12	0.5 (6)	C14—C13—C18—C19	−179.5 (2)
C10—C11—C12—C7	−0.2 (5)	C13—C18—C19—C20	−178.8 (3)
C10—C11—C12—C13	179.1 (3)	C18—C19—C20—C21	−175.8 (3)
C8—C7—C12—C11	0.4 (4)	C2—C3—C22—N	−32 (24)
C6—C7—C12—C11	−179.6 (3)	C4—C3—C22—N	147 (24)

## References

[bb1] Allen, F. H., Kennard, O., Watson, D. G., Brammer, L., Orpen, A. G. & Taylor, R. (1987). *J. Chem. Soc. Perkin Trans. 2*, pp. S1–19.

[bb2] Enraf–Nonius (1994). *CAD-4 EXPRESS* Enraf–Nonius, Delft, The Netherlands.

[bb3] Harms, K. & Wocadlo, S. (1995). *XCAD4* University of Marburg, Germany.

[bb4] Jiang, P., Zhao, D. D., Yang, X. L., Zhu, X. L., Chan, J. & Zhu, H. J. (2012). *Org. Biomol. Chem.* doi:10.1039/C2OB25120E. 10.1039/c2ob25120e 22572762

[bb5] North, A. C. T., Phillips, D. C. & Mathews, F. S. (1968). *Acta Cryst.* A**24**, 351–359.

[bb6] Omer, K. M., Ku, S. Y., Chen, Y. C., Wong, K. T. & Bard, A. J. (2010). *J. Am. Chem. Soc.* **132**, 10944–10952. 10.1021/ja104160f 20681728

[bb7] Sheldrick, G. M. (2008). *Acta Cryst.* A**64**, 112–122. 10.1107/S0108767307043930 18156677

